# Cytological Identification of a Rhabdoid Variant of Pancreatic Neuroendocrine Neoplasm

**DOI:** 10.1111/cyt.70118

**Published:** 2026-08-27

**Authors:** Miyu Okuda, Keishi Mizuguchi, Tatsuya Mori, Urara Okawa, Hiroko Ikeda

**Affiliations:** ^1^ Department of Diagnostic Pathology Kanazawa University Hospital Kanazawa Japan

## Abstract

Pancreatic neuroendocrine tumors (PanNETs) are rare epithelial neoplasms with endocrine differentiation. Although most PanNETs exhibit typical cytological features, uncommon morphological variants occasionally occur. We report the first case of a rhabdoid‐type PanNET diagnosed solely by cytology. A 48‐year‐old woman was incidentally found to have a pancreatic head mass. Endoscopic ultrasound‐guided fine‐needle aspiration cytology revealed tumor cells with eccentric nuclei, granular chromatin, and prominent eosinophilic intracytoplasmic inclusions, which were highlighted by Giemsa staining. Immunocytochemistry demonstrated strong cytokeratin positivity within the inclusions along with the expression of neuroendocrine markers. This case underscores that rhabdoid‐type PanNET can be diagnosed through the cytological identification of distinctive intracytoplasmic inclusions and confirmed by immunocytochemistry. Awareness of this rare phenotype is essential for accurate diagnosis and improved clinicopathological understanding of PanNET variants.

## Graphical abstract

Intracytoplasmic inclusions observed in rhabdoid‐type neuroendocrine tumours are evident on Giemsa staining and show strong cytokeratin (AE1/AE3) positivity on immunocytochemistry.

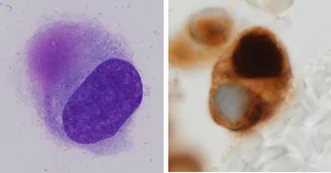

This report describes the cytological features of a rare rhabdoid variant of pancreatic neuroendocrine neoplasm and highlights the diagnostic challenges associated with this entity. The tumour exhibited characteristic intracytoplasmic inclusions and atypical morphology that may lead to misinterpretation in cytological specimens. Recognition of these features is important for accurate diagnosis and appropriate clinical management.

## 1 Introduction

Pancreatic neuroendocrine tumours (PanNETs) are rare epithelial neoplasms with endocrine differentiation, accounting for approx. 2% of all pancreatic tumours, typically occurring between ages 40 and 60. While most arise in the pancreatic tail, they can also develop in the head [1]. PanNETs are usually slow‐growing and carry a favourable prognosis, even with metastasis [1]. They are classified as functioning or non‐functioning tumours based on hormone secretion, though cytomorphology is similar in both types [1]. Classical PanNETs show tumour cells arranged in rosettes or singly, with granular cytoplasm, thin nuclear membranes, eccentrically placed nuclei, and ‘naked nuclei’ appearance. Chromatin displays a fine‐to‐coarse granular (‘salt‐and‐pepper’) pattern [2].

Although classic features allow for relatively straightforward diagnosis, atypical morphologies can pose significant diagnostic challenges. Reported variants include rhabdoid, oncocytic, lipid‐rich, and papillary/solid‐pseudopapillary neoplasm–like types [3]. Rhabdoid PanNETs are characterised by tumour cells with a rhabdoid or signet‐ring–like appearance and by prominent paranuclear cytoplasmic protein inclusions [4, 5]. In addition to the entities typically considered in the differential diagnosis of PanNETs, such as acinar cell carcinoma (ACC) and solid pseudopapillary neoplasm (SPN), rhabdoid PanNETs must also be differentiated from plasmacytoma, rhabdomyosarcoma, signet‐ring cell carcinoma, and rhabdoid tumour [3].

We report a rare case of PanNET with rhabdoid morphology, characterised by eosinophilic intracytoplasmic inclusions, and describe the cytological features observed with both Papanicolaou (Pap) and Giemsa stains along with immunocytochemical findings, demonstrating that the histologic subtype can be inferred from cytology alone.

## 2 Case Report

### 2.1 Clinical Summary

A 48‐year‐old woman was referred to our hospital for evaluation and treatment after a 6 cm pancreatic head tumour was incidentally detected on CT following a fall. She was otherwise asymptomatic with no significant medical history. Dynamic CT revealed a hypervascular peripheral region with heterogeneous enhancement and central non‐enhancing areas during the pancreatic parenchymal phase. PET‐CT showed FDG uptake at the tumour periphery, findings inconsistent with pancreatic ductal adenocarcinoma, raising suspicion for a high‐grade malignancy such as adenosquamous carcinoma. Endoscopic ultrasound‐guided fine‐needle aspiration (EUS‐FNA) suggested PanNET, so pancreaticoduodenectomy was performed. No metastases were identified in the lymph nodes or other organs. The patient is alive with no evidence of recurrence or metastasis two years after surgery.

### 2.2 Cytological Findings

Endoscopic ultrasound‐guided fine‐needle aspiration (EUS‐FNA) was performed using a 22‐gauge needle, and rapid on‐site evaluation (ROSE) was conducted at the bedside. Although only mild atypia was noted on ROSE, the presence of cells suspicious for malignancy was confirmed; therefore, the procedure was terminated. A cell block was prepared from the needle rinse fluid.

Atypical cells, ranging from isolated to small clusters, were observed in a haemorrhagic background. These small cells had abundant cytoplasm and round to oval eccentrically located nuclei with a rhabdoid appearance. The chromatin showed a fine to coarse granular, salt‐and‐pepper pattern, and some cells had nucleoli (Figure 1A). Distinct intracytoplasmic inclusions appeared pink and were clearly demarcated from the surrounding cytoplasm on Giemsa staining (Figure 1B). In contrast, on Pap staining, the inclusions exhibited a coloration similar to that of the cytoplasm, making their clear identification difficult. Based on the nuclear features, the scattered distribution of rhabdoid cells together with the imaging findings, PanNET was the favoured diagnosis.

**FIGURE 1:**
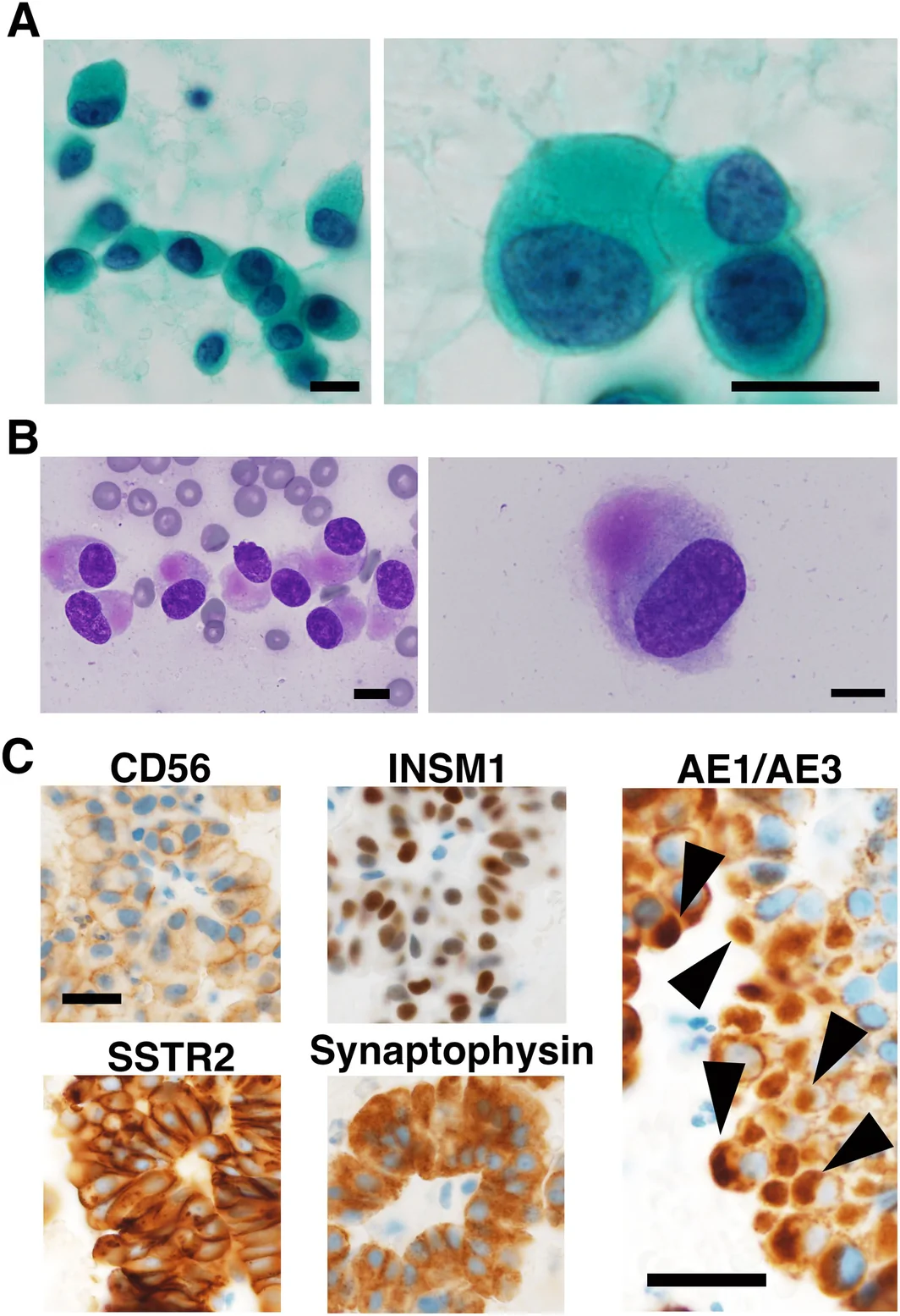
(A) Left: Papanicolaou staining: Scattered tumour cells and small clusters are observed against a haemorrhagic background. Scale bar: 10 μm. Right: High‐power view: The tumour cells show abundant cytoplasm and eccentrically located round to oval nuclei, exhibiting rhabdoid morphology with salt‐and‐pepper chromatin. Scale bar: 10 μm. (B) Left: Giemsa staining: Well‐defined intracytoplasmic inclusions are evident within tumour cells. Scale bar: 10 μm. Right: High‐power view. (C) Immunocytochemical findings on the cell block: CD56‐positive, INSM1‐positive, SSTR2‐positive, and Synaptophysin‐positive. Scale bar: 20 μm. The AE1/AE3 was strongly positive in the cytoplasmic inclusions (arrow heads). Scale bar: 20 μm.

Immunocytochemistry on the cell block demonstrated positivity for neuroendocrine markers, including CD56, INSM1, SSTR2, and synaptophysin, supporting the diagnosis of PanNET. AE1/AE3 showed strong positivity corresponding to the intracytoplasmic inclusions (Figure 1C), a characteristic staining pattern that enabled the specific diagnosis of rhabdoid PanNET. These findings indicate that even in the absence of histological specimens, cytology alone can provide sufficient evidence for a definitive diagnosis.

### 2.3 Histological Findings

The resected pancreas contained a 5‐cm dark reddish, firm‐elastic mass in the pancreatic head (Figure 2A). Histologically, erythrocyte and fibrin accumulation corresponded to the grossly dark reddish areas (Figure 2B). Haematoxylin and eosin staining showed tumour cells arranged in rosette‐like patterns around capillary‐sized vessels. The tumour cells were round, with abundant cytoplasm and eccentrically located round to oval nuclei. The nuclei exhibited salt‐and‐pepper chromatin, and the cytoplasm contained pale eosinophilic intracytoplasmic inclusions (Figure 2C). Although the tumour was large, no necrosis was observed.

**FIGURE 2:**
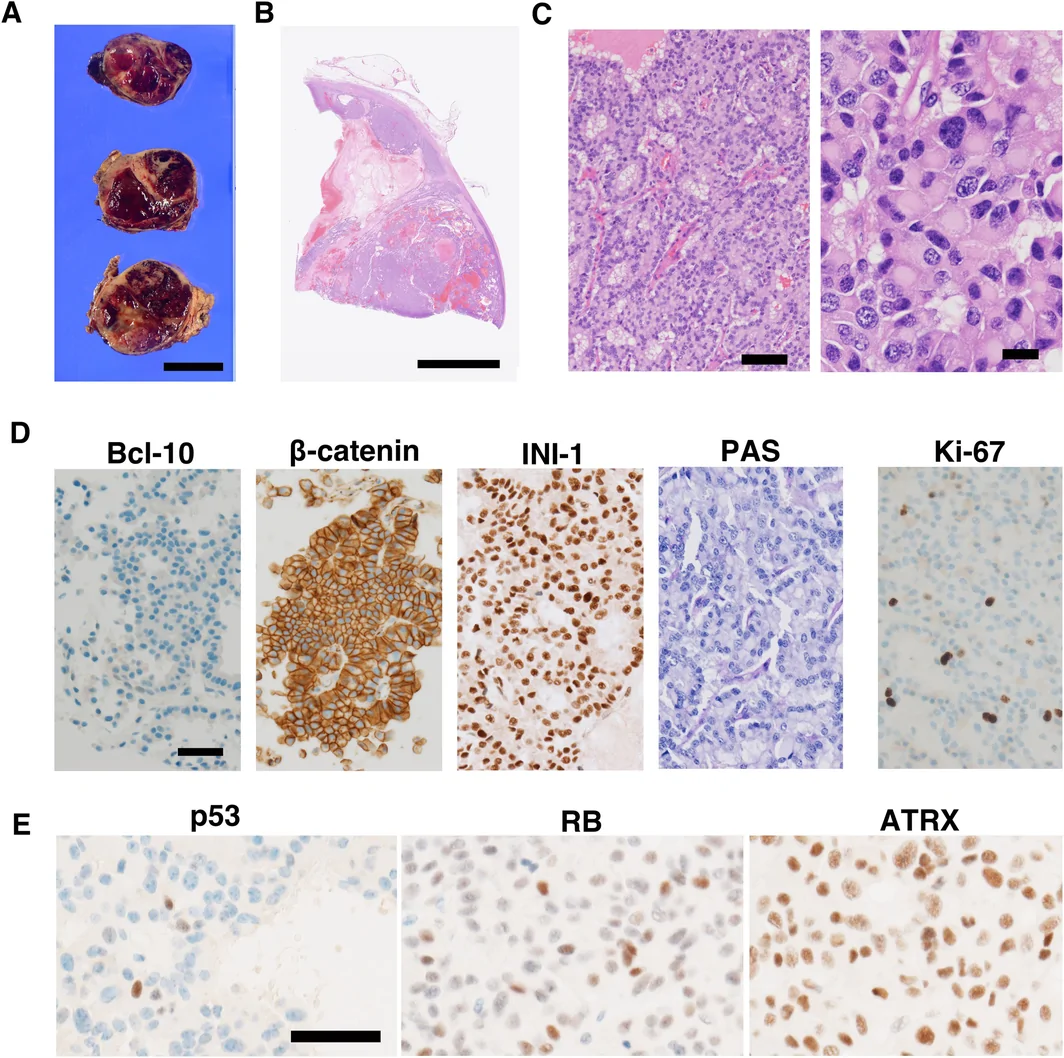
(A) Gross appearance: 5‐cm dark reddish mass in the pancreatic head. Scale bar: 30 mm. (B) Macro view of haematoxylin and eosin (H&E) staining: Abundant erythrocytes and fibrin corresponding to the dark reddish regions. Scale bar: 10 mm. (C) Left: Low‐power view of H&E staining showing rosette‐like arrangements of tumour cells around capillaries. Scale bar: 100 μm. Right: High‐power view showing round tumour cells with eccentrically located nuclei, abundant cytoplasm, salt‐and‐pepper chromatin, and pale eosinophilic intracytoplasmic inclusions. Scale bar: 20 μm. (D) Bcl‐10 negativity (excluding ACC), absence of nuclear β‐catenin expression (excluding SPN), retained INI1 expression (excluding rhabdoid tumour), and negative PAS staining (excluding adenocarcinoma). Ki‐67 index: 6%. Scale bar: 50 μm. (E) p53 and RB wild type, retained ATRX expression. Scale bar: 50 μm.

The tumour cells were positive for neuroendocrine markers, consistent with the immunocytochemistry findings (Table 1) (Figure 2D,E). Additional immunostaining was performed to exclude ACC, SPN, rhabdoid tumour, and adenocarcinoma, and all were ruled out based on the results. The Ki‐67 index was approximately 6% in hotspot areas. The Ki‐67 index was assessed by manual counting in both FNA and surgical specimens.

**TABLE 1:** The clinicopathologic features of reported cases of neuroendocrine tumour of the pancreas with rhabdoid features.

case	Age	sex	location	Size (cm)	Ki‐67 index	Positive IHC markers	Metastases	Treatment	Outcome	References
1	50	F	Head	4.5	6%	Synaptophysin, focal chromogranin, AE1/AE3, CAM5.2, vimentin	No metastasis	pancreaticoduodenectomy + lymphadenectomy	No follow‐up available	[4]
2	35	M	NA	3.2	< 5%	AE1/AE3, CAM5.2, gastrin, insulin, focal somatostatin, vasoactive intestinal peptide, α‐subunit	3 lymph nodes metastasis; peripancreatic soft tissue invasion	NA	Alive without recurrence (2 years)	[5]
3	55	M	NA	11	< 5%	AE1/AE3, CAM5.2	9 lymph nodes metastasis; peripancreatic soft tissue invasion	NA	Alive without recurrence (5 years)	[5]
4	45	F	NA	5	< 5%	AE1/AE3, CAM5.2, α‐subunit	No metastasis	NA	No follow‐up available	[5]
5	41	F	NA	8	< 5%	AE1/AE3, CAM5.2, gastrin, glucagon	1 lymph node metastasis	NA	No follow‐up available	[5]
6	67	F	NA	6	< 5%	AE1/AE3, CAM5.2, α‐subunit, ACTH	1 lymph node metastasis	NA	Dead (2 years after diagnosis)	[5]
7	53	M	Head	5.7	3%	Synaptophysin, AE1/AE3, CAM5.2, SMARCB1 (INI1), BAF155, BAF170, ARID1A, weakly BRG‐1, CK19, EMA, NCAM, weakly gastrin, wild type p53	4 lymph nodes positive; Multiple Liver matastasis	Enucleation + lymphadenecotomy + partial hepatectomy	Alive without recurrence (4.5 years)	[6]
8	60	M	Head	NA	< 1%	Synaptophysin, chromogranin, AE1/AE3, CAM5.2	Bilobar liver metastasis	Radiation + chemotherapy + alcohol injection	Dead (73 months after diagnosis)	[7]
9	69	F	Head??	3.4	20%–25%	CD56, INSM1, synaptophysin, focal chromogranin, AE1/AE3, focal S‐100	Multiple Liver matastasis	Pancreaticoduodenectomy + partial hepatectomy	No follow‐up available	[8]
10	37	M	Tail	3.4	ND	Synaptophysin, chromogranin, AE1/AE3, gastrin	Periaortic lymph nodes metastasis	Partial pancreatectomy + lymphadenectomy	Alive without recurrence (2 years after surgery)	[9]
11	55	F	Head	2.5	ND	Synaptophysin, chromogranin, AE1/AE3	Multiple liver metastasis	Pancreaticoduodenectomy + partial hepatectomy	Dead (peritonitis, 1 month after surgery)	[9]
12	58	M	Head	4	ND	Synaptophysin, chromogranin, AE1/AE3	No metastasis	Pancreaticoduodenectomy	Alive without recurrence (4 years after surgery)	[9]
13	79	F	Tail	4.5	ND	Synaptophysin, chromogranin, AE1/AE3	No metastasis	Distal pancreatectomy + splenectomy	Alive without recurrence (5 years after surgery)	[9]
Present case	48	F	Head	6	6%	CD56, INSM1, synaptophysin, SSTR2, focal chromogranin, AE1/AE3, SMARCB1 (INI1) retain, ATRX retain, wild type p53, wild type Rb	No metastasis	Pancreaticoduodenectomy + lymphadenectomy	Alive eith recurrence (2 years after surgery)	

Abbreviations: NA, not available; ND, not detected.

Taken together, the immunohistochemical profile confirmed a definitive diagnosis of PanNET G2 with a rhabdoid phenotype.

## 3 Discussion

We presented a rare case of rhabdoid PanNET, a neoplasm with distinctive cytokeratin‐positive intracytoplasmic inclusions requiring careful morphological and immunostaining evaluation for accurate diagnosis and clinical management.

Rhabdoid‐type PanNETs are characterised by distinctive intracytoplasmic eosinophilic inclusions critical to morphological identification, comprising keratin filaments known as ‘cytokeratin aggresomes’ [4]. ‘Rhabdoid’ refers to cells containing eosinophilic inclusions that resemble immature skeletal muscle cells (rhabdomyoblasts) [4, 6], although they are not true rhabdomyoblasts and have no relation to muscle tissue [4]. The keratin‐based nature of these inclusions is critical for differential diagnosis by immunostaining as discussed later. In our case, cytokeratin‐positive intracytoplasmic inclusions were the decisive feature in a presumptive cytological diagnosis of the rhabdoid type.

Rhabdoid‐type PanNETs show cytologic and histologic overlap with other neoplasms. They may be misinterpreted as SPN, plasmacytoma, signet‐ring cell carcinoma, or rhabdomyosarcoma, due to the diffuse growth pattern and cytologic morphology [3]. Immunostaining is essential for differentiation. Rhabdoid‐type PanNETs are positive for neuroendocrine markers including chromogranin A, synaptophysin, and INSM1, and importantly, the intracytoplasmic inclusions stain positive for cytokeratin AE1/AE3 [6, 7, 10]. In contrast, SPNs are positive for nuclear β‐catenin and CD10; rhabdomyosarcomas are positive for desmin and MyoD1; plasmacytomas are positive for CD79a and CD138; and signet‐ring cell carcinomas are negative for neuroendocrine markers but positive for mucin staining [10]. Rhabdoid tumours show loss of nuclear staining for SMARCB1 (INI1) [10]. Selection of an appropriate immunohistochemical panel is critical for accurate diagnosis.

In this case, PanNET G3 and neuroendocrine carcinoma (NEC) were excluded based on the Ki‐67 index. Cytologically, the tumour showed a relatively uniform appearance with only mild atypia, and no features of poor differentiation such as marked nuclear atypia or necrosis, making NEC unlikely. Furthermore, p53 had a wild‐type expression pattern, with no findings suggestive of TP53 gene alteration, and RB expression was retained in the nuclei, indicating no clear evidence of RB pathway inactivation. ATRX expression was also preserved in the nuclei, with no suggestion of ATRX abnormality [10, 11]. Alterations in TP53 and RB are rare in PanNETs, and when present, raise a differential diagnostic concern for high‐grade NEC. As no findings suggestive of such abnormalities were identified in this case, the tumour was considered to fall within the spectrum of a typical PanNET from a molecular standpoint as well. The patient exhibited no hormone‐related specific symptoms, and the tumour was diagnosed as a non‐functioning pancreatic neuroendocrine tumour (PanNET). In previous reports, non‐functioning cases have been predominant, and no clear correlation has been observed between morphological features and hormone secretion [5, 9].

Regarding diagnostic considerations, it is important not to rely solely on the Ki‐67 labeling index when making a diagnosis. Moreover, in fine‐needle aspiration (FNA) specimens, limited sampling may result in insufficient cellularity, making accurate classification difficult. Therefore, an accurate diagnosis requires comprehensive evaluation of morphological findings, in order to avoid misclassification.

The cytological identification of intracytoplasmic inclusions is a key feature for recognizing rhabdoid‐type PanNETs, and Giemsa staining is particularly useful for this purpose [8]. These inclusions can resemble mucin in signet‐ring cell carcinoma, requiring careful differentiation. In Giemsa staining, both rhabdoid cell inclusions and the acidic cytoplasmic mucin of signet‐ring cells appear as pink, globular structures (Figure 1B). However, in Pap staining, rhabdoid cell inclusions stain light green and show indistinct borders from the surrounding cytoplasm, making them difficult to recognise (Figure 1A). In contrast, signet‐ring cell carcinoma demonstrates a relatively well‐defined interface between mucin and cytoplasm in Pap staining, facilitating distinction. Thus, the combined use of Pap and Giemsa staining enhances the recognition of rhabdoid cells and underscores the diagnostic utility of cytology in identifying rhabdoid‐phenotype PanNETs. Distinguishing classical‐type PanNETs from ACC and SPN can also be challenging. However, ACC and SPN exhibiting rhabdoid morphology have not been reported to date, suggesting that the presence of rhabdoid morphology in cytological specimens of pancreatic masses may be a useful feature for excluding ACC and SPN.

Rhabdoid morphology in PanNETs has been associated with aggressive behaviour in some cases, including increased tumour size, lymph node metastasis, local invasion, and early recurrence [3]. According to previous studies, metastases are present in approximately half of cases at diagnosis, occasionally involving multiple liver metastases [7, 9]. However, Chetty and Asa reported a case confined to the pancreas without metastasis [4]. Similarly, in our case, despite a tumour size of 6 cm, the lesion remained localised (Table 1). Thus, rhabdoid morphology does not uniformly indicate aggressive behaviour. Although PanNETs with rhabdoid morphology are rare and their prognostic significance remains uncertain, reports of unfavourable outcomes underscore the clinical importance of recognizing and documenting this morphology when present [3].

In conclusion, rhabdoid‐type PanNETs can be diagnosed cytologically with immunocytochemical support, though further case series are needed given the rarity of this phenotype.

## Author Contributions

**Miyu Okuda:** conceptualisation (equal); investigation (equal); writing – original draft (lead). **Keishi Mizuguchi:** conceptualisation (equal); methodology (lead); writing – review and editing (lead). **Tatsuya Mori:** investigation (equal). **Urara Okawa:** investigation (equal). **Hiroko Ikeda:** supervision (lead).

## Funding

The authors have nothing to report.

## Ethics Statement

The case discussed in this manuscript does not include patient identifying information, nor does it report a new study that required IRB approval. Under the Ethical Guidelines for Medical and Biological Research Involving Human Subjects (Ministry of Health, Labour and Welfare, Japan), case reports are not considered research requiring ethical review. Therefore, ethical approval was not necessary for this study.

## Conflicts of Interest

The authors declare no conflicts of interest.

## Data Availability

The data that support the findings of this study are available from the corresponding author upon reasonable request.

## References

[1] G. Martignoni , F. Pedica , M. Falconi , D. Antonello , G. Malpeli , and A. Scarpa , “Endocrine Neoplasms of the Pancreas: Pathologic and Genetic Features,” Archives of Pathology & Laboratory Medicine 133, no. 3 (2009): 350–364. 10.5858/133.3.350 19260741

[2] S. Y. Zee , S. N. Hochwald , K. C. Conlon , M. F. Brennan , and D. S. Klimstra , “Pleomorphic Pancreatic Endocrine Neoplasms: A Variant Commonly Confused With Adenocarcinoma,” American Journal of Surgical Pathology 29, no. 9 (2005): 1194–1200. 10.1097/01.pas.0000164370.81132.25 16096409

[3] Y. Xue and M. D. Reid , “Well‐Differentiated Pancreatic Neuroendocrine Tumor: Does Morphologic Variant Matter?,” Archives of Pathology & Laboratory Medicine 150 (2025): e123–e130. 10.5858/arpa.2025-0034-RA 40438920

[4] R. Chetty and S. L. Asa , “Pancreatic Endocrine Tumour With Cytoplasmic Keratin Whorls. Is the Term ‘Rhabdoid’ Appropriate?,” Journal of Clinical Pathology 57, no. 10 (2004): 1106–1110. 10.1136/jcp.2004.018309 PMC1770450 15452172

[5] S. Serra , S. L. Asa , and R. Chetty , “Intracytoplasmic Inclusions (Including the So‐Called ‘Rhabdoid’ Phenotype) in Pancreatic Endocrine Tumors,” Endocrine Pathology 17, no. 1 (2006): 75–81. 10.1385/ep:17:1:75 16760583

[6] T. Miyazaki , S. Aishima , M. Fujino , et al., “Neuroendocrine Tumor of the Pancreas With Rhabdoid Feature,” Virchows Archiv 473, no. 2 (2018): 247–252. 10.1007/s00428-018-2398-x PMC6096768 29938394

[7] J. Shia , R. A. Erlandson , and D. S. Klimstra , “Whorls of Intermediate Filaments With Entrapped Neurosecretory Granules Correspond to the ‘Rhabdoid’ Inclusions Seen in Pancreatic Endocrine Neoplasms,” American Journal of Surgical Pathology 28, no. 2 (2004): 271–273. 10.1097/00000478-200402000-00018 15043320

[8] J. J. Fite , S. Z. Ali , and C. J. VandenBussche , “Fine‐Needle Aspiration of a Pancreatic Neuroendocrine Tumor With Prominent Rhabdoid Features,” Diagnostic Cytopathology 46, no. 7 (2018): 600–603. 10.1002/dc.23892 29359517

[9] M. D. Perez‐Montiel , W. L. Frankel , and S. Suster , “Neuroendocrine Carcinomas of the Pancreas With ‘Rhabdoid’ Features,” American Journal of Surgical Pathology 27, no. 5 (2003): 642–649. 10.1097/00000478-200305000-00007 12717248

[10] Board WHOCoTE, International Agency for Research on C , Digestive System Tumours, 5th ed. (International Agency for Research on Cancer, 2019).

[11] Z. Ankers , L. S. Hellgren , A. Stenman , J. Zedenius , and C. C. Juhlin , “Distinguishing Neuroendocrine Tumor Grade 3 From Neuroendocrine Carcinoma in Gastroenteropancreatic High‐Grade Neoplasms,” Endocrine Oncology 5, no. 1 (2025): e250065. 10.1530/EO-25-0065 PMC12728940 41450443

